# The Capacity of Artificial Intelligence in COVID-19 Response: A Review in Context of COVID-19 Screening and Diagnosis

**DOI:** 10.3390/diagnostics12122943

**Published:** 2022-11-25

**Authors:** Dilber Uzun Ozsahin, Nuhu Abdulhaqq Isa, Berna Uzun

**Affiliations:** 1 Department of Medical Diagnostic Imaging, College of Health Sciences, Sharjah University, Sharjah P.O. Box 27272, United Arab Emirates; 2 Operational Research Center in Healthcare, Near East University, TRNC Mersin 10, Nicosia 99138, Turkey; 3 Department of Biomedical Engineering, Near East University, TRNC Mersin 10, Nicosia 99138, Turkey; 4 Department of Biomedical Engineering, College of Health Science and Technology, Keffi 961101, Keffi Nasarawa State, Nigeria; 5 Department of Statistics, Carlos III Madrid University, 28903 Getafe, Madrid, Spain; 6 Department of Mathematics, Near East University, TRNC Mersin 10, Nicosia 99138, Turkey

**Keywords:** COVID-19 diagnosis, AI in COVID-19, CT images, CXR images, COVID-19 screening

## Abstract

Artificial intelligence (AI) has been shown to solve several issues affecting COVID-19 diagnosis. This systematic review research explores the impact of AI in early COVID-19 screening, detection, and diagnosis. A comprehensive survey of AI in the COVID-19 literature, mainly in the context of screening and diagnosis, was observed by applying the preferred reporting items for systematic reviews and meta-analyses (PRISMA) guidelines. Data sources for the years 2020, 2021, and 2022 were retrieved from google scholar, web of science, Scopus, and PubMed, with target keywords relating to AI in COVID-19 screening and diagnosis. After a comprehensive review of these studies, the results found that AI contributed immensely to improving COVID-19 screening and diagnosis. Some proposed AI models were shown to have comparable (sometimes even better) clinical decision outcomes, compared to experienced radiologists in the screening/diagnosing of COVID-19. Additionally, AI has the capacity to reduce physician work burdens and fatigue and reduce the problems of several false positives, associated with the RT-PCR test (with lower sensitivity of 60–70%) and medical imaging analysis. Even though AI was found to be timesaving and cost-effective, with less clinical errors, it works optimally under the supervision of a physician or other specialists.

## 1. Introduction

COVID-19 early screening and diagnosis can be challenging for differentiating from lung and other respiratory infections, due to common shared dominant symptoms [1], such as fever, flu, cough/catarrh, etc. Apart from scarcity and time consumption (six to eight hours [2]), the polymerase chain reaction (PCR) test, which is considered the standard for COVID-19 diagnosis [3], has been extensively reported to have high false-positive rates [4,5]. Hence, there was sporadic and high community infection spread, due to lack of early diagnosis [6,7]. Relatively, COVID-19 can also be diagnosed using serology, PCR, nasal/throat swab testing, and medical imaging [8,9,10]. Any pandemic of international concern requires swift response to decode the virus and contain the spread through early screening and diagnosis. AI has a record of accomplishment for being fast, cost effective, and accurate when applied in medical processes to assist physicians. AI uses computer algorithms to stimulate human-like intelligence to accomplish specific tasks without being actively encoded with additional commands [11,12]. In medicine, AI aims to increase and decrease diagnostic accuracy errors, respectively [13]. In the COVID-19 pandemic, AI has been applied in several areas, including screening and decision making via predictive and analytical models [14]; however, its application was very limited [7] during the onset of the pandemic. After a while, AI gradually developed to meet the demand of rapid and precise early detection and diagnosis, which became the most focused area of AI application during the pandemic [15]. This study investigates the capacity of AI in the early COVID-19 screening, detection, and diagnosis of COVID-19 by comprehensively surveying the current related literature using the guidelines of preferred reporting items for systematic reviews and meta-analyses (PRISMA).

## 2. Methodology

**Figure 1:**
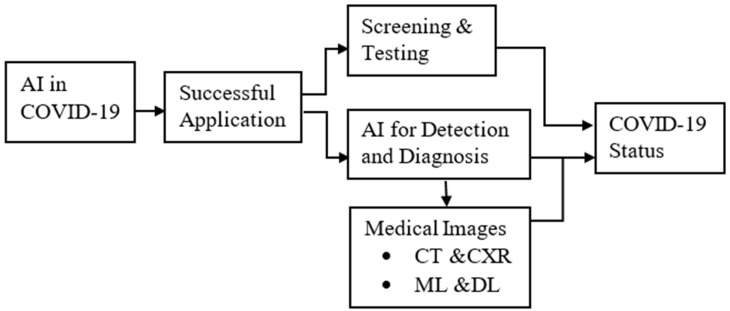
Layout of the paper.

The approach used for this review does not contain any clinical data (no prospective or retrospective data) of patients; hence, there was no need to get an ethical consent to conduct the study. Data were collected from journals that published articles as open sources. The following considerations were taken: article information, such as publication date, author’s names, methodology, AI models, dataset, and the overall outcome of the study (sensitivity, specificity, and accuracy). Articles for review were selected for the years 2020, 2021, and the current year 2022, making it a comprehensive literature review of AI roles in COVID-19 screening and diagnosis. The keywords focused on included COVID-19, COVID-19 diagnosis, AI in COVID-19, CT images, CXR images, and COVID-19 screening. The framework of the study is shown in Figure 1.

Selected articles for these studies were chosen based on application of AI in COVID-19 screening, diagnosis, or both. This included the use of medical imaging, PCR testing, or other laboratory examinations. Lastly, all selected articles used English language in the manuscripts. Some studies that reviewed the significance of AI in COVID-19 diagnosis included [7,15,16,17,18,19,20,21,22,23,24,25,26,27] and to mention but a few.

## 3. AI for COVID-19 Diagnosis

AI algorithms can interpret large and high dimension biomedical data, especially for complex medical diagnoses that normal statistical approaches cannot handle [28], and data sources that can be used to create models that aid in prediction are disconnected [29]. There are several AI approaches, the success of which depend on factors such as context, quality, and context of dataset [30]. For example, it is more suitable to perform image segmentation by convolutional neural networks (CNN) [31]. These techniques include those that handle the medical imaging data used for detection and diagnosis [32] and other clinical data for prediction, contact tracing, and COVID-19 social control [33,34,35]. It is significant to combine other clinical data alongside imaging data in a single dataset to achieve the bigger aim of diagnosing COVID-19 by AI [36].

### 3.1. Areas of Successful Application

AI used for screening and diagnosing COVID-19 involves models that auto control, recognize, segment, and classify COVID-19 data.

#### 3.1.1. AI in Screening and Testing

**Figure 2:**
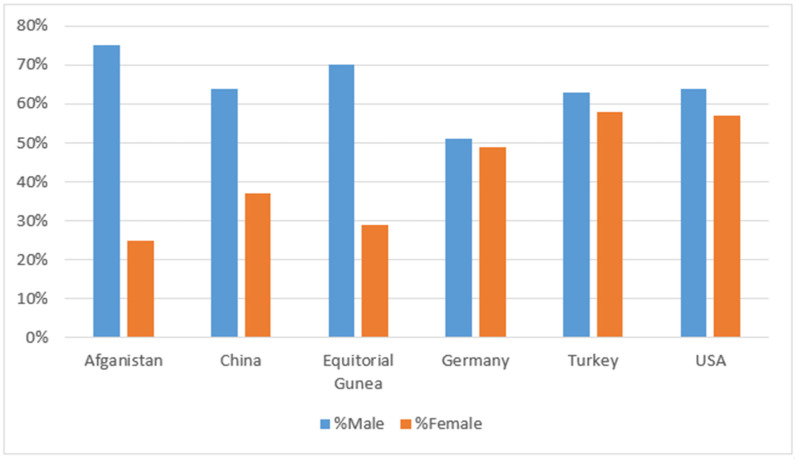
Cross-country COVID-19 infection, showing gender as a factor that can affect screening (El-Rashidy et al., 2022) [23].

Screening can be used to predict the infection status of individuals using a combination of vital factors, which among many benefits, helps physician effectively manage limited resources, especially during the pandemic. AI models used for screening of COVID-19 ranges from simple tools such as rule-based scoring systems to complicated tools such as deep learning [32,37]. Medical images can also be combined with non-imaging data to identify biomarkers and predict disease risk factors [38,39]. Several factors are beneficial for screening COVID-19 suspects, in order to prevent/contain sporadic and community transmission. This is also helpful in contact tracing to sever the transmission chain. Factors such as age, gender, ethnicity, symptoms, and existing health issues, etc., can be helpful in screening for possible COVID-19 infection within a population. Age, gender, and other demographic information can help in screening COVID-19 [15]. Figure 2 shows gender as a factor affecting COVID-19. Reports have shown that the disabled population and immune-compromised individuals are at high risk of COVID-19 infection, as well as elderly people and individuals with underlying health issues [40,41] therefore, AI can help these groups.

The nucleic acid amplification (NAATs) and antigen tests have also been improved by AI. RT-PCR tests are the traditional and dominant COVID-19 diagnostic tools that use specimen samples from oropharyngeal and nasopharyngeal swabs or blood [23]. Screening can include the use of clinical symptoms (fever, cough, fatigue, hemoptysis, low white blood cells, and migraine) that are plagued with challenges, such as time wastage and high level of false positivity of results. AI has the ability to improve the performance of traditional COVID-19 testing and the results. In fact, imaging data can be combined with non-imaging data from electronic health records (EHRs) or elsewhere for identifying biomarkers and predicting disease risk factors [38].

Researchers have incorporated these factors in AI to automatically screen for COVID-19. Ref. [42] identified some discriminant clinical characteristics, such as hyposmia (loss of smell) and hypogeusia (loss of taste), that were incorporated into telemedicine for mass screening. Ref. [43] proposed an AI model that utilized the demographic, clinical data, and chest images of patients to screen and detect COVID-19. However, the model was only applied for a very limited patient sample. Furthermore, early in the pandemic, body temperature was a major screening tool that was used in the public to screen for COVID-19. This tool can also be combined with other vital information that can identify COVID-19 status [15]. Saha et al. [15] developed an AI model that was able to identify the vital factors linked to COVID-19. This was possible by incorporating these factors to provide an accurate prognosis that can assist physicians with swifter diagnosis. This model also improves the stress mounted on physicians, since it helps screen the individuals that require the most help.

#### 3.1.2. AI in COVID-19 Detection and Diagnosis

##### Medical Images for AI COVID-19 Diagnosis

Imaging modalities, such as computed tomography (CT), magnetic resonance imaging (MRI), and X-ray, are valuable tools used in detecting and quantifying COVID-19 [19]; however, chest x-ray (CXR) and CT images are most widely used globally [44]. AI models for COVID-19 diagnosis using medical images usually include pre-scan/processing image acquisition and diagnosis. AI works to mainly segment or classify (or both) medical images. Prepossessing includes noise reduction and segmenting images to find a region of interest (ROI), while image classification extracts details or features from ROI, which is the basis for diagnosis [22]. Generally, medical images provide excellent pathological details; however, these details can be ignored, due to the lack of automation, which allows for both quantitative and qualitative assessments [24]. The details that are ignored are a thin line between a false positive or negative. According to Mauger et al. [45] and Bai et al. [46], imaging data can provide important traits that can be obtained through assessing the qualitative and quantitative structural changes within the image, thus making it time effective. Several AI models accurately distinguish COVID-19 from pneumonia, based on the image features contained in both CXR and CT [47].

**Figure 3:**
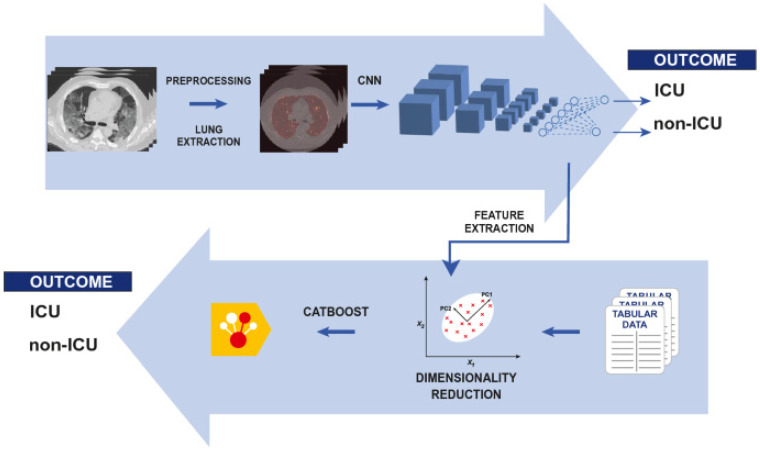
Severity and progression of COVID-19 virus reveal by AI through medical images and other clinical data [39].

AI can also reveal severity or progression of COVID-19 and predict clinical outcome [48] as illustrated in the study of Chieregato et al. [39], shown in Figure 3. Chieregato et al. [39] developed a hybrid model of machine and deep learning to classify patients into non-ICU and ICU (intensive care admission or death). The study also included other clinical data to provide clinical decision support. Pan et al. [49] utilized AI on chest CT images to identify and categorize COVID-19 severity and progression into four stages: the early (≤4 days), progressive (5–8 days), peak (9–13 days), and absorption (14 ≥ days) stages. Darapaneni et al. [50] identified COVID-19 severity through CXR, using bounded boxes with RCNN mask for ROI and improved accuracy.

##### CT and X-ray Medical Images for COVID-19 Diagnosis

**Figure 4:**
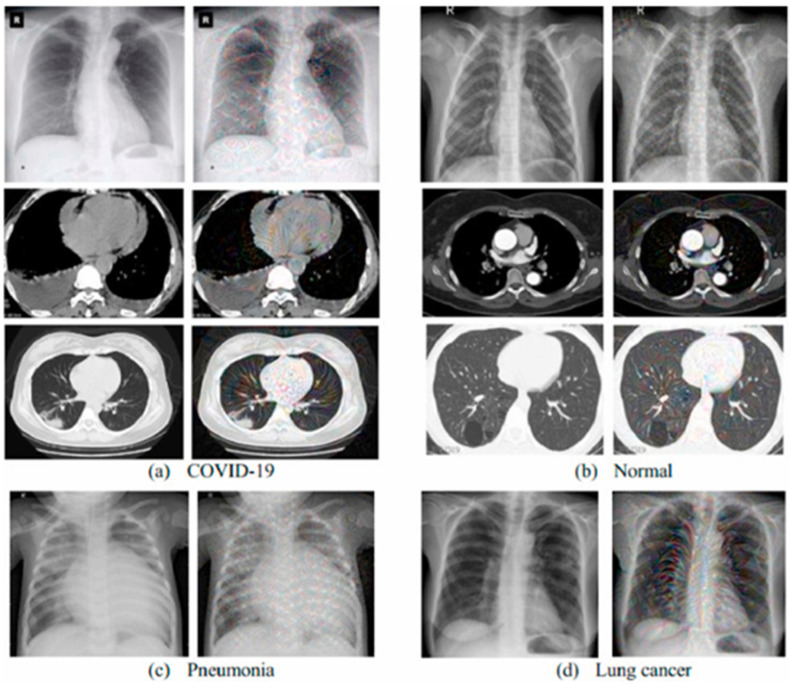
CT and X-ray Medical Images utilized for Diagnosing COVID-19 and distinguishing from other diseases (Ramdani et al., 2021) [17]. (**a**,**b**) shows CT images of COVID-19 of normal patient, while (**c**,**d**) shows CXR images of Pneumonia and lung cancer.

The CT and X-ray images have been globally available to physicians, especially during the COVID-19 pandemic. This is because of their fast acquisition, aiding the diagnostic process [51]. CT is recorded to have the quickest turn-around time, rather than the PCR test, and provides specific information of the pathogen of interest [15]. CT images identify ground-glass opacity (GGO) around the sub-pleural region [6,8,52,53,54] and have a better qualitative measurement of lesions [32]. AI utilizes the CT lung images of patients with fever-like symptoms to determine their COVID-19 status. It distinguishes between bacterial infection, pneumonia, and COVID-19 [55]. Although CXR images are less informative, compared to CT images, due to the lower sensitivity, the popularity and availability of X-ray imaging facilities means that it is widely used for diagnosis. AI with CXR identifies ground-glass opacity (GGO) around the subpleural region, providing evidence and classification of COVID-19 severity. AI applied to chest x-ray generally involves image preprocessing, classification, and evaluation of the model, and does not usually involve segmentation step, such as in in chest CTs, because the ribs are projected onto other tissues in CXR, making it difficult [56]. Figure 4 shows the CT (Figure 4a,b) and X-ray (Figure 4c,d) medical images used for diagnosing and distinguishing COVID-19 from other diseases.

There are several studies in 2020, 2021, and 2022 that utilize both CXR and CT chest images to identify or distinguish between COVID-19 and other respiratory illnesses using AI models. Al-Waisy et al. [57] enhanced the contrast of CXR images and significantly reduced the image noise with adaptive histogram equalization and a Butterworth band pass filter. The results of their study showed 99.99%, 99.98%, and 100% accuracy, sensitivity, and specificity, respectively. The noise in the X-ray images used by Horry et al. [58] were also reduced using a simple VGG19 pre-train deep learning model to detect COVID-19. The recorded accuracy was 83%. Similar to Al-Waisy et al. [57], Lawton et al. [59] used contrast and adaptive histogram equalization on CT images of the lung to diagnose COVID-19 using a transfer learning approach.

Gilanie et al. [60] detected COVID-19 using chest radiology images with CNN. The study achieved mean accuracy, specificity, and sensitivity of 96.68 %, 95.65 %, and 96.24%, respectively. For 2022, Kumar and Mahapatra [18] proposed a novel deep CP-CXR model. The model was used to perform two experiments: a binary and multi-class classification between COVID-19 and normal patients. After inputting CXR into the model, the study achieved 100%, and 98.57% accuracy for binary and multi-class classification, respectively. In [61] Sajun et al. showed that with a small proportion of labeled data, the FixMatch model was able to performed almost the best supervised techniques. Xue et al. [62] identified structural abnormalities in CXR images using a COVID-19 deep learning model that uses several layers of convolution networks. The classification achieved a final accuracy of 97.67%. Musha et al. [63], Canario et al. [64], El-Dahshan et al. [65], and Amin et al. [66] all used CT and CXR images in deep learning models to diagnose COVID-19 infection. These studies obtained accuracies of 97.9%, 98%, 99%, 99.3%, and 96.6%, respectively.

Several AI-based studies focused on radiological images with the same aim of diagnosing COVID-19, but they differed in the algorithms they applied for segmenting and classifying ROI. Some of these algorithms used for segmentation included U-Net, U-Net++, and V-Net, etc., while the ResNet and CNN models with inception were mostly used for classification [30]. The authors Khan et al. [67], and Dhiman et al. [68] utilized CXR alongside other clinical data with the aim of detecting and differentiating between COVID-19 patients from others. However, these studies used different approaches and algorithms to reach the same aim. Khan et al. [67] developed a join-fusion AI system (with accuracy of 97%) that combines CXR images with other clinical data that are passed through multiple layers until classification is reached to determine the COVID-19 status of a patient. Dhiman et al. [68] identified COVID-19 infection using a proposed hybrid AI model derived from comparative analysis of 11 distinct deep learning structures, in order to establish an improved image feature extraction. The study recorded the highest accuracy when Resnet-101, with the J48 algorithm decision tree, and the hyena optimizer were comparatively utilized together.

Ieracitano et al. [69] developed a novel convolutional neural network (CovNNet) that utilizes fuzzy preprocessing principles on CXR images to determine patients’ COVID-19 status early. After preprocessing and application into multiple convolution layers, the proposed CovNNet was able to determine (classify) COVID-19 status within a short time (50 s) and an accuracy of 80.9%. Nayak et al. [70] reached a conclusion that ResNet-34 model performs better than other networks, after classifying and recognizing images in different CNN pre-trained models. Unfortunately, the model of the study does not take multi-class classification into consideration.

Nishio et al. [71] developed a CNN model utilizing a dataset categorized into COVID-19, pneumonia, or normal CXR images. The model consisted of transfer learning and the widely used EfficientNet. After validating and classifying (using ensemble approach) the dataset, the result of the final ensemble was compared to the clinical judgement of six experienced radiology professionals (10 months to 15 years). The CNN model of the study outperformed the six radiologists combined. In another study, Yildirim et al. [30] extracted features from images to determine the status into three categories: COVID-19, viral pneumonia, or normal. Two datasets were used, which were inputted in the system with feature maps from MobileNetV2, EfficientNet0, and DarkNet53. The study further tested the performance of several classifiers to diagnose COVID-19 and other disorders. The SVM classifier performed the best in all the datasets in the study, with mean accuracies of 99.05% and 97.1% in the first and second datasets, respectively. Sharma et al. [72] developed a novel fusion-based convolutional neural network approach for the classification of COVID-19 from CXR images. The model was abbreviated as COVDC-Net and utilized two datasets: first (COVID-19, normal, and pneumonia) and second (COVID-19, healthy, bacterial pneumonia, and viral pneumonia images). After successfully running the classification, the study achieved total accuracies of 96.48% and 90.22% for the first and second datasets, respectively. Xu et al. [44] used a real-time COVID-19 detection model on CXR images in edge computing. The model challenges the status quo by using a lightweight CNN with an increased dataset capacity by DCGAN. Classification was carried out for MobileNetV2, ResNet18, and VGG19, with functional accuracies of F1 scores of 82%, 87%, and 83%, accordingly. A similar study by Rajawat et al. [13] utilized a lightweight CNN model (C-COVIDNet) to classify CXR images as COVID-19, pneumonia, or normal, achieving an accuracy of 97.5%.

##### Machine Learning (ML) and Deep Learning (DL) for COVID-19 Diagnosis

Both ML and DL have been applied in medical processes prior to COVID-19′s emergence. During the COVID-19 pandemic, they have been actively employed in studies for the diagnosis, treatment, and prognosis of the virus. ML can handle huge datasets of novel viruses and their genomes, creating room for more comprehension of the mysterious COVID-19 virus [19]. ML has a wide range of techniques in AI that include supervised, unsupervised, semi-supervised, and reinforcement learning [12]. Supervised learning uses labels to assist in image classification, while unsupervised learning identifies patterns in the unlabeled data [73]. On the other hand, DL is a sub-class of ML that has gained attention in medical applications over the years for decoding medical imaging details [74]. The DL method is characterized by multiple network layers that work individually and collectively to extract points from a dataset. The lower network layers mostly function to identify details for images edges/lines, while higher network layers identify more specialized details. The layers work successively to achieve a common output [75].

Studies that used deep learning on either X-ray or CT images (or both) to detect COVID-19 include Panwar et al. [76], Gilanie et al. [60], and Amin et al. [66], etc. DL was used alongside thoracic CT images to create a complete AI system that detects COVID-19 and measures severity and disease progression [77]. A DL model (XR-CAPS) was created by Darji et al. [78] to segment and extract points in CXR images to predict COVID-19. The model fuses a UNet model and capsule network for segmentation and feature extraction, respectively. The study utilized 896 participants, categorized in to healthy, pneumonia, COVID-19 positive. After running the model, the study achieved accuracy, sensitivity, and specificity scores of 93.2%, 94%, and 97.1%, respectively. López et al. [79] used a similar method as Darji et al. [78], achieving an 86.30% accuracy score after attempting to differentiate between COVID-19 and other non-pneumonia cases.

A DL technique that was developed to generate synthetic radiographic data for aiding detection of COVID-19 features in radiographs is the data augmentation of radiographic images (DARI) algorithms [80,81]. DL models have shown accuracy scores of up to 96.3% in anterior–posterior radiographs, which are within the acceptable scores that the reduce errors of false positives and negatives [81]. DL models were found to be capable of distinguishing between the closely related symptoms of COVID-19 and flu, a task that has not been easy for physicians [82]. This capacity is especially significant for developing countries, where limited diagnostic resources have been identified as a major challenge during the pandemic.

A comprehensive dataset that utilized chest CT images, symptoms, contact history, and lab tests was used in a joint CNN model that was developed by Mei et al. [43]. The CNN model is a rapid diagnostic tool for COVID-19. Other AI models that were developed for distinguishing symptoms of other diseases that are similar to that COVID-19 include AD3D-MIL model [83], Ensemble of Bagged Tree [84], and AutoML Cloud Vision [85], and they were proven to have acceptable performance. Xu et al. [44] developed a model that classify CT images in categories of influenza-A viral pneumonia (IAVP), COVID-19, and non-respiratory disorders. The model consists of 3D CNN for segmentation and a ResNet-based model for classification. The dataset consists of the COVID-19 diagnosed CT scans confirmed by RT-PCR test. The data were tested and validated and achieved a 86.7% accuracy. Ahuja et al. [86] obtained a sensitivity score of 92.21% and specificity score of 98.50 after attempting to detect COVID-19 using their CNN model. The dataset included radiographic images of the lung, which were extracted, and the opacities were specified. Utilizing multi-objective differential evolution and convolutional neural (MODE-CNN) networks, Singh et al. [87] diagnosed COVID-19 through chest CT images. Lastly, a study by Alom et al. [88] created an AI system (COVID_MTNet) to diagnose COVID-19. The system used improved inception recurrent residual neural network (IRRNN) and NABLA-3 network models for the purpose of detecting and localizing ROIs in CXR and CT images.

A summary of some of the studies reviewed in this study is provided in Table 1. The table provides an overview of the studies reviewed in the study, taking into consideration the year of publication, the reference details of the authors and their affiliation, the AI model used in the study, the dataset used in the study, and the accuracy outcomes of the model.

## 4. Result: COVID-19 Status

**Figure 5:**
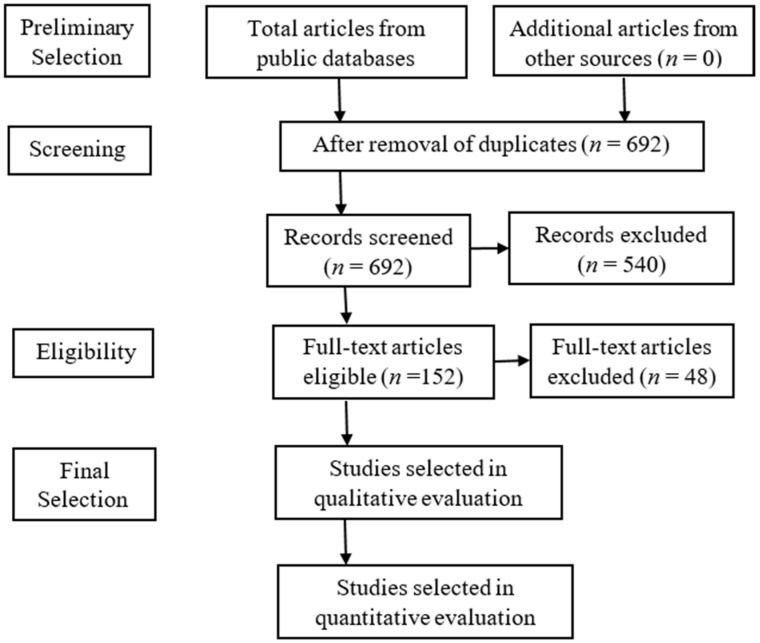
Block diagram of selection criteria by PRISMA guidelines for the study.

Preliminarily, 720 articles were collected from public domains and sampled to meet selection criteria. PRISMA guidelines were used for the selection criteria, as shown in Figure 5. After the PRISMA procedure, only 152 studies were eligible and selected (final) for qualitative evaluation. The dataset used in most of these studies were mostly medical images of CT and CXR images. Other studies took non-imaging data into consideration, such as PCR tests and patient characteristics (such as symptoms, age, gender, etc.).

After a comprehensive review, the results found that AI contributed immensely to improving COVID-19 screening and diagnosis. Some proposed AI models were shown to have comparable (sometimes even better) clinical decision outcomes, compared to experienced radiologists, in screening/diagnosing COVID-19. Some of the studies have shown their AI models to have the capacity to learn and comprehend high-dimensional features that are usually difficult or impossible for humans to handle (such as texture and wavelet points), with the excellent distinguishing capacity of COVID-19 from other respiratory diseases.

Additionally, AI has the capacity to reduce physician work burden and fatigue and reduce the problems of several false positives, which are associated with RT-PCR tests (with lower sensitivity of 60–70%) and medical imaging analyses. Compared to low scores of human and RT-PCR tests, most of the studies have shown an average score of about 90% for the accuracy, sensitivity, and specificity of the models. Despite these capabilities of AI, there is limited study directly comparing human and AI performance. Some of the studies used radiological data to diagnose, predict, and estimate the patterns of COVID-19. Some of the benefits of AI identified in our review include, but are not limited to, time saving, improving clinical outcomes (accuracy, specificity, and sensitivity), swift responses, and reduced physician workload. It has also been shown to provide real-time diagnostic capacity when used with medical images. Even though AI was found to be timesaving and cost-effective, with less clinical errors, it works optimally under the supervision of a physician or other specialists. Because of AI, the screening and diagnosis of COVID-19 was improvedved to identify and prevent sporadic community spread and high community infection. Several models were developed for academic articles, while others were practically applied in healthcare and for community mitigation purposes.

## 5. Conclusions and Future Recommendation

The COVID-19 virus is still within us, and we must continue to develop approaches that will help aid the response and fight to mitigate the pandemic. AI has provided a significant improvement in the COVID-19 pandemic response; however, there is still more to be accomplished, especially in the aspect of screening and diagnosis. It is important to note that AI is still at a preliminary stage, so there is much that it can offer in the future as it continues to evolve. There should also be thorough central quality control for testing the validity of any developed AI system for medical and healthcare applications, in order to guarantee patient safety. This is because the review has not found a consensus for national and international guidelines or policies that regulate the adoption and implementation of AI systems for COVID-19. Global uniformity in clinical data should be encouraged. This is because there is variability in tests and diagnostics globally, thus affecting the labeling process. There is a challenge of an available domain that scientists can provide physicians with developed AI systems for COVID-19. This domain can make it accessible for physicians, to improve their performance. Although it is still important to regulate the use of AI systems for patient safety, some of the developed AI systems maybe too complicated for physicians to comprehend, explain, interpret, or apply. In fact, most of the studies have limitations of small datasets, relevant clinical comparators, and external validation. These create biases that makes the developed models practically inapplicable. Therefore, the recommended future research is to make their models meet these concerns. Furthermore, COVID-19 symptoms were shown to be similar to that of other diseases, especially respiratory diseases; therefore, there is need for it, and it is recommended that both medical images and non-medical imaging data not be used for diagnosis, in order to reduce and prevent errors.

This study has provided a review from several studies from 2020, 2021, and 2022, focusing on the screening and diagnosis aspects of AI’s contribution to fighting COVID-19. As seen in the review, some proposed AI models were shown to have a comparable (sometimes even better) clinical decision, compared to experienced radiologists, in diagnosing COVID-19. AI models identified vital features or details that can easily be ignored by human intelligence. Some of the studies reviewed in this study achieved an accuracy as high as 100%, which is contrary to the human intelligence that usually includes errors that may affect final clinical decisions. Thus, AI has the capacity to reduce physician work burden and fatigue and reduce the problems of several false positives, associated with RT-PCR tests (with lower sensitivity of 60–70%) and medical imaging analysis. From the review, we also found AI to be time saving and cost effective, with less clinical errors. Despite these capabilities, AI works optimally under the supervision of a physician or other specialists. The pandemic highlights the huge role AI has in mitigating not only the current pandemic, but also future pandemics that are predicted to occur in this century. Hence, the workload for physicians and other health care workers can be significantly reduced, and the effective use of resources can be encouraged. The major limitation of this study is that it was not possible to include all relevant articles for the years 2020, 2021, and 2022. Additionally, only articles in English were included, thus ignoring several important articles that may have been published in other languages. Despite these facts, this study is significant because it highlights the role and emerging trend of AI in COVID-19 screening and diagnosis, which can help aid or improve medical decision making during the COVID-19 pandemic. The COVID-19 pandemic also reveals the significant role of engineers in assisting medical physicians; therefore, the continuous collaboration between the two fields (especially biomedical engineering) should be encouraged. This will give rise to novel innovations that will take into consideration time, cost, and limited resources, among others.

## Figures and Tables

**Table 1:** Summary of some reviewed papers showing dataset, AI model, and study outcome.

Year	Reference	Type	Model	Dataset	Accuracy
2020	[42]	Screening	-	image and clinical data	
	[43]	screening	-		
	[50]	Images	RCNN	CXR	-
	[57]	Images	VGG19	CXR	99.99%
	[58]	images	VGG19	CXR	83%
	[44]	Images	3D CNN	CT slices	86.7%
2021					
	[79]	Images	Deep learning	CT	86.30%.
	[86]	Images	CNN	CT	92.21%
2022	[18]	Images	CP-CXR	CXR	100%
	[62]	Image	Deep learning	CXR	97.67%
	[67]	Image	join-fusion AI system	CXR	97%
	[69]	Image	CovNNet	CXR	80.9%
	[78]		XR-CAPS/UNet	CXR	93.2%

## Data Availability

Data is provided within the manuscript.
